# The Morphology of Peri-Implantitis Bone Defects: A Retrospective Study on Periapical Radiographs

**DOI:** 10.1155/2024/4324114

**Published:** 2024-04-30

**Authors:** Alice Alberti, Benedetta Morandi, Caterina Frascolino, Nicolo Cavalli, Luca Francetti, Stefano Corbella

**Affiliations:** ^1^Department of Biomedical, Surgical and Dental Sciences, Università degli Studi di Milano, Milan, Italy; ^2^IRCCS Ospedale Galeazzi—Sant'Ambrogio, Milan, Italy; ^3^Private Practioner, Geneve, Switzerland

## Abstract

**Objectives:**

The aims of this study were to assess the morphologic features of peri-implant defects, as measured on 2D intraoral radiographs, and to investigate the possible correlation between such morphology and other parameters related to the position and characteristics of the implant or the implant-supported prosthesis.

**Materials and Methods:**

Implants with peri-implantitis were included in this retrospective study. Data collected were related both to the patients and to the position/characteristics of the implants and the implant-supported prosthesis. Measurements of the morphologic defects were performed by two operators on digitalized intraoral periapical radiographs. *Results and Conclusion*. In total, 73 implants in 27 patients were included. The measurements of the periapical radiograph suggested that the most common defect conformation was crateriform, with both intraosseous and horizontal components. An inverse correlation was found between the extension of the peri-implant lesion and the time between the radiographic assessment and the implant placement. The total lesion area was strongly correlated to oral hygiene levels. No correlations between lesion extension and smoking, diabetes, history of periodontal were found. In conclusion, the results from this 2D radiographic study showed the prevalence of crateriform peri-implant defects, with a hygiene-correlated extension, perceptible on the mesial and distal aspects; 3D imaging could be used when available for further research and clinical investigation.

## 1. Introduction

The morphology of peri-implant lesions influences both the choice of the surgical approach and the implant prognosis. It was demonstrated that the clinical success of surgical regenerative therapy of peri-implantitis also depends on the morphological features of the peri-implant lesions: in particular, contained circumferential peri-implant defects (also referred to as Class Ie) [1] registered better results in terms of probing depth (PD) reduction and clinical attachment level gain as compared to other types of defects [2].

In 2007, Schwarz et al. [1] classified the configurations of the peri-implantitis defects on the basis of clinical examination in Class I (intraosseous defects) and Class II (horizontal bone loss). Class I defects were then divided into five different configurations (a, b, c, d, and e) in relation to the position of the bone defect compared to the implant body. The authors found that the most common defect configuration was the circumferential one (Class Ie), with a frequency of 55.3% in humans [1]. Similarly, Serino et al. [3] showed that 66% of the implants showed a similar extent of bone resorption throughout the implant circumference, while in 34% of them, bone loss was larger on the buccal aspect than on other surfaces.

Another study found that 25% of the defects were circumferential combined with a buccal dehiscence, while 30% of the defects were Class Ie [4]. Probably, the fact that peri-implantitis evolves in a more aggressive and severe way in the buccal sites is due to the proximity of the dental implants to the cortical bone and the bone architecture [4–6]. In a more recent study, Monje et al. [7] proposed a new classification of peri-implantitis defects according to their morphology and severity, assessed through tridimensional radiographic examination. As for defect morphology, three major categories were identified, namely intraosseous (Class I), supracrestal (Class II), and combined defects (Class III). Class I defects were further classified as A (with buccal dehiscence), B (2- or 3-wall defect), or C (circumferential defect). The severity of the peri-implant lesion was graded as slight if not exceeding 25% of the implant length, moderate if varying between 25% and 50%, and advanced if more than 50% of the implant length [7].

The primary aim of the present radiographic study was to assess the morphologic features of peri-implant defects, as measured on 2D intraoral radiographs, which are used as a standard routine for monitoring peri-implant health. Secondarily, the study aimed at investigating the possible correlations between such morphology and other parameters related to the position and characteristics of the implant or the implant-supported prosthesis. The null hypothesis is that such parameters do not affect the peri-implant defect morphology.

## 2. Materials and Methods

This retrospective study was conducted in accordance with the declaration of Helsinki on human studies, and it was part of a research project that was approved by the Internal Board of the IRCCS Istituto Ortopedico Galeazzi (number of approval L4153/2020). The study was reported here according to the STROBE statement.

### 2.1. Study Population

All patients treated with dental implants and attending the Department of Dentistry of IRCCS Istituto Ortopedico Galeazzi, Milan, from January 2005 to September 2021, were screened for inclusion, and patients with a diagnosis of peri-implantitis were considered.

Peri-implantitis was defined following the consensus report of the 2017 World Workshop on the Classification of Periodontal and Peri-implant Diseases and Conditions [8]. In particular, when previous clinical and radiographic examinations were present, peri-implantitis was defined as follows:

- Presence of bleeding and/or suppuration on gentle probing;
- Increase in PD with reference to previous clinical examinations;
- Radiographic loss of the peri-implant bone.

Conversely, when the patient did not attend the regular follow-ups and no clinical or radiographic data were present, a case of peri-implantitis was defined following these parameters:

- Presence of bleeding and/or suppuration on gentle probing;
- PD ≥6 mm;
- Peri-implant bone level ≥3 mm apical to the most coronal portion of the intraosseous part of the implant.

All data were treated anonymously following the current privacy norms.

### 2.2. Eligibility Criteria

The inclusion criteria were as follows:

- Patients treated with implant-supported single-crowns, fixed partial dentures, or full-arch prostheses;
- Age ≥18 years;
- Diagnosis of peri-implantitis;
- Complete radiographic and clinical documentation, together with data about smoking habits and systemic disease.

The following exclusion criteria were applied:

- Intraoral radiographs with insufficient quality to perform radiographic measurements;
- Intraoral radiographs which were not performed with the parallel technique with Rinn intraoral sensor holder with paralleling system rings;
- Radiographic control performed with radiographic examinations other than intra-oral periapical radiograph;
- Patients who did not attend to the maintenance program for more than 1 year.

### 2.3. Data Collection

The following parameters were recorded: age, sex, gender, smoking habit, history of periodontitis, diabetes, drugs assumption, presence of systemic diseases, frequency of professional oral hygiene recalls, hygiene levels according to the Simplified Oral Hygiene Index (John G. Greene D.M.D., M.P.H.  ^*∗*^, Jack R. Vermillion M.P.H. The Simplified Oral Hygiene Index. The Journal of the American Dental Association Volume 68, Issue 1, January 1964, Pages 7–13).

The following data related to the implant treatment were retrieved from clinical records: age of the patient at the time of implant placement, length and diameter of the implant, implant brand, whether guided bone regeneration (GBR) was performed and, eventually, with which material, type of implant-supported prosthesis, time of provisional prosthesis placement, and time of definitive prosthesis placement.

**Figure 1:**
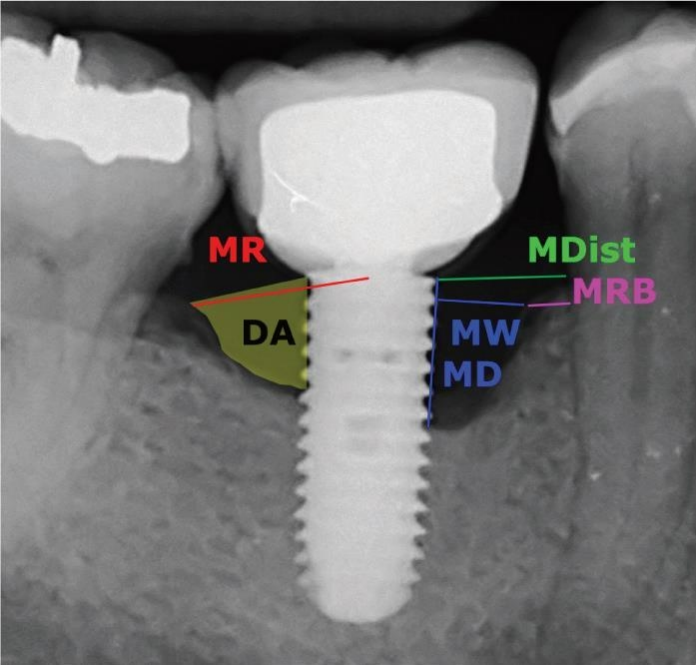
Graphic representation showing the radiographic measurements (MR: maximum radius; MDist: mesial distance; MD: mesial depth; MW: mesial width; MRB: residual mesial bone; and DA: distal area).

**Figure 2:**
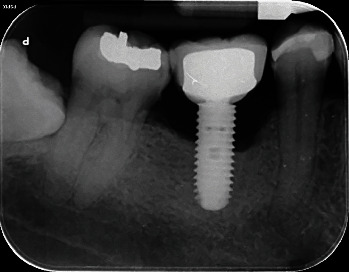
Radiograph of one of the included implants with peri-implantitis: the most common defect conformation in the present study was crateriform, with both intraosseous and horizontal components.

The following measurements were then performed by two operators on digitalized intraoral periapical radiographs (Table 1; Figures 1 and 2):

- Maximum radius of the peri-implant lesion, as measured from the center of the implant platform to the most distant coronal bone crest level;
- Mesial and distal depth of the lesion;
- Mesial and distal width of the lesion;
- Mesial and distal distance between the implant and the adjacent tooth or adjacent implant;
- Residual bone crest level (mesial and distal);
- Area of the lesion on the mesial side and area of the lesion on the distal side in mm^2^.

The operators who performed the measurements were calibrated on the first five radiographs.

### 2.4. Statistical Analysis

Descriptive statistical analysis was presented in terms of means and standard deviations for continuous variables, together with a 95% confidence interval and minimum–maximum range. Pearson correlation was used to evaluate the presence of a statistical correlation between the investigated variables. A linear regression test was performed to assess the effect of the studied parameters on dependent variables. For all the analyses, *α* was set at 0.05.

## 3. Results

A total of 73 implants belonging to 27 patients were included in the study. Thirteen subjects were males (accounting for 30 implants), while 14 were females (43 implants), and the mean age at surgery was 56.5 ± 7.84 years. Most of the implants were positioned in native bone, while in 20 cases, GBR procedures were performed before or simultaneously with implant placement. As for implant position, the majority of the implants were placed in the posterior areas, both mandibular and maxillary, being 2.5 and 2.4 the most frequent positions (10.9% and 9.6%, respectively), followed by site 4.6 (8.2%). The type of prosthesis placed was AllOn4® in more than half of the cases, partial prosthesis on two or three implants in 23 cases, and single crowns in 9 cases. Most of the implant-supported prostheses were screw-retained. Among all implants, four failed and were extracted.

As for parameters related to well-known peri-implantitis risk factors, it was registered that 32 out of 73 diseased implants belonged to 12 smoking patients, three implants to one subject with diabetes, and 45 implants to 17 subjects with a history of periodontitis. As for oral hygiene level, patients had poor, fair, and good plaque control, respectively, in nearly 35%, 45%, and 20% of the cases (implant-level), with a recall frequency of 6 months in almost 60% of the cases.  Table 2 summarizes patient- and implant-related data, while Table 1 shows the results of the registered parameters related to the peri-implant lesion morphology.

**Figure 3:**
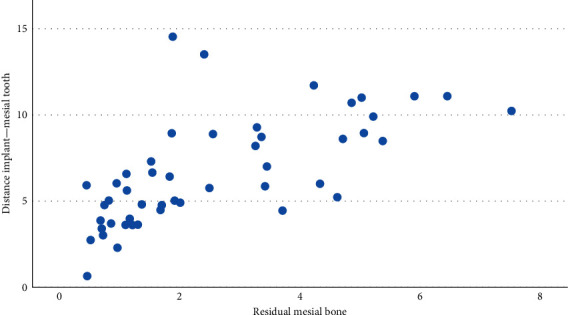
Graphic showing the positive correlation between the mesial residual bone with the distance of the mesial element (calculated in mm). The direction of the correlation shows that a higher distance from the mesial element is correlated to wider residual bone.

**Figure 4:**
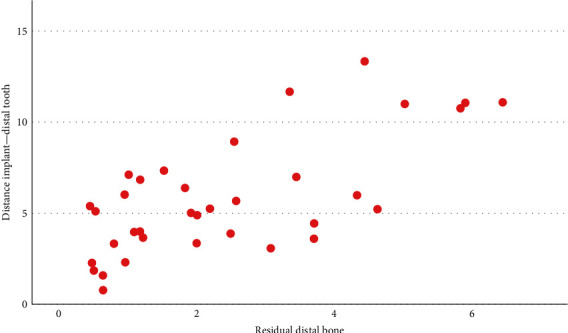
Graphic showing the positive correlation between the distal residual bone with the distance of the distal element (calculated in mm). Similarly to Figure 3, higher distances from the adjacent implant or tooth are associated with wider residual bone.

**Figure 5:**
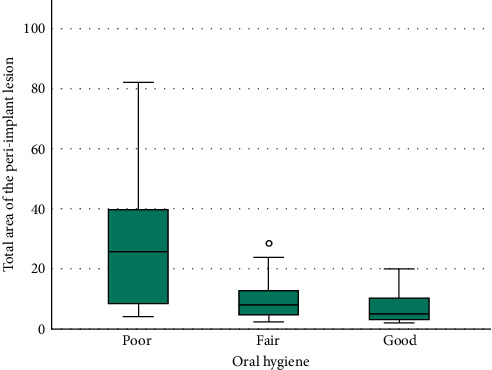
Box plot graphic showing the influence of oral hygiene on the extension of the peri-implant lesion (mm^2^). Despite the high variability of measures, the figure clearly shows that poor oral hygiene is associated with larger lesions.

**Figure 6:**
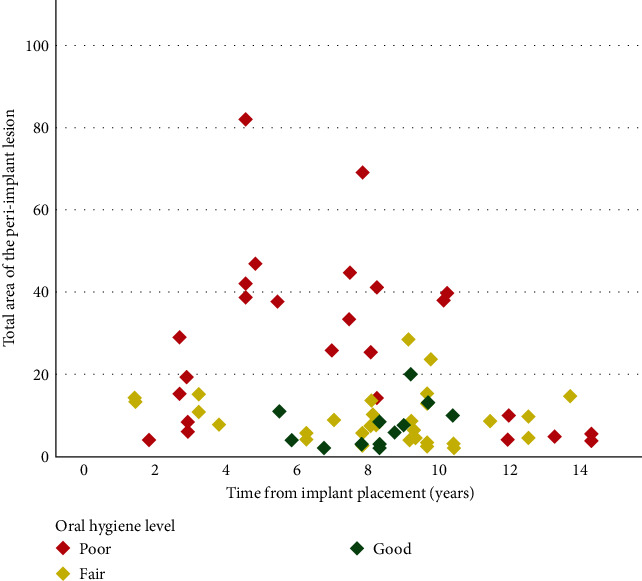
Graphic showing the correlation between the total area of the peri-implant lesion (mm^2^) and the time elapsed from implant placement. In this graphic too, results were stratified by oral hygiene level in order to better visualize its effect on the progression of peri-implant pathology.

Strong correlations were highlighted between some of the evaluated parameters, in particular the mesial and distal height of the lesion (*r* = 0.88, *p*  < 0.001); the distal area and the mesial area (*r* = 0.792, *p*  < 0.001); the height of the distal lesion and the mesial area (*r* = 0.719, *p*  < 0.001); the width of the mesial lesion with the distal area (*r* = 0.412, *p*  < 0.001) and the distal height (*r* = 0.381, *p*=0.001); the distal width with mesial area (*r* = 0.424, *p*  < 0.001), mesial height (*r* = 0.411, *p*  < 0.001), and mesial width (*r* = 0.437, *p*  < 0.001). The distance of the distal element is correlated with the width of the distal lesion (*r* = 0.492, *p* = 0.001); the distance of the mesial element with the mesial residual bone (*r* = 0.657, *p*  < 0.001) (Figure 3) and the width of the medial lesion (*r* = 0.409, *p*=0.003); the distal residual bone with the distance of the distal element (*r* = 0.697, *p*  < 0.001) (Figure 4). A strong correlation was also found between the total area of the peri-implant lesion and the implant brand 3i; however, the analysis was performed only on three implants from this brand, belonging to the same patient. The total lesion was strongly correlated to oral hygiene levels, with an inverse direction (Figure 5). This was confirmed by Student's *t* test (*p*  < 0.001). An inverse statistically significant correlation was found between the extension of the peri-implant lesion and the time between the radiographic assessment and the implant placement (Figure 6). Correlations between the lesion extension and various risk factors such as smoking, diabetes, and history of periodontal disease were also explored, but no correlation was found in the investigated population.

## 4. Discussion and Conclusion

A careful interpretation of the results regarding the registered radiographic parameters is needed to make some clinical considerations. While some of the registered linear correlations between radiographic parameters are simply coherent with the geometry of the lesion (e.g., the height of the mesial lesion with the mesial area), some others are specifically associated with the morphologic conformation: in particular, linear correlations between mesial and distal parameters can be translated clinically in peri-implant defects involving contemporarily the mesial and distal side of the implant. For example, there was a strong correlation between the distal and mesial areas, the distal height and the mesial area, the mesial height and the distal height, the width of the mesial lesion with the distal area and the distal height, and, conversely, the distal width and the mesial area, mesial height, and mesial width. These results suggest the most common defect conformation was crateriform, with both intraosseous and horizontal components, which is in accordance with the results of Schwarz et al. [1], who most frequently observed Ie defects (55.3%), which were often combined with Class II defects. Data related to the mean total area of the lesions present a high standard deviation, which can be explained by the presence of a single patient with a huge peri-implant lesion, which led to the extraction of the implant itself. The results regarding oral hygiene show how peri-implantitis is a plaque-correlated disease. Actually, the total area of the peri-implant lesion was correlated to oral hygiene levels. This must be considered clinically, since larger lesions present a challenging complexity to treatment and less chance of successful treatment outcome. Therefore, it is of paramount importance to frequently monitor patients with poor hygiene levels, in order to timely diagnose peri-implantitis before the development of advanced lesions.

Although this study includes only diseased implants and is not designed to identify factors associated with peri-implantitis, some considerations can be made on the analyzed sample: first, only 18.9% of the implants belong to patients with good oral hygiene. Moreover, two thirds of the diseased implants were in the posterior areas, which is more difficult to access for oral hygiene maneuvers. Similarly, more than 80% of the prostheses supported by implants affected by peri-implantitis were of the AllOn4® type and of the partial multi-unit type: it can be hypothesized that the presence of the prosthetic superstructure may have prevented patients from cleaning properly around the neck of the implant.

As for risk factors like diabetes, smoking, and history of periodontitis, the present study revealed a lack of correlation. Nevertheless, this could be due to the size of the sample and the insufficient variability of these parameters throughout the sample. Actually, there is consistent evidence from longitudinal studies and longitudinal and cross-sectional studies that both smoking and a history of periodontitis constitute a risk factor/indicator of peri-implantitis. For example, a 10-year cohort study by Karoussis et al. [9] found that 18% of all implants in smokers developed peri-implantitis, while only 6% of implants in nonsmokers were affected. Similarly, in one study evaluating 218 patients up to 14 years after implant therapy, implants placed in patients with a history of periodontitis were significantly more likely to develop peri-implantitis compared to non-periodontal patients, with an OR = 5 [10]. Karoussis et al. [9] and Koldsland et al. [11] reported similar results after examining 109 subjects with 1–16 years of follow-up (OR = 6). One large 10-year longitudinal study by Roccuzzo et al. [12] followed 101 patients with implants and classified them as (1) periodontally not compromised, (2) moderately compromised, and (3) severely compromised. The authors reported that both the frequency of implant sites with PD ≥6 mm (2%, 16%, and 27%, respectively) and bone loss ≥3 mm (5%, 11%, and 15%, respectively) differed significantly between groups. An inverse statistically significant correlation was found between the extension of the peri-implant lesion and the time between the radiographic assessment and the implant placement, which again can be explained by the small size of the sample. In fact, peri-implantitis is a worsening pathology, which, therefore, worsens with the passage of time. An example of this is the study by Fransson et al. [13], who evaluated 182 patients with a total of 419 implants who presented with progressive bone loss. For these implants, bone levels were assessed using intraoral radiographs obtained at the 1-year examination and a follow-up period of 5–23 years (mean: 11.1 years). The mean bone loss was 1.7 mm, and the cumulative rates of implants with ≥1, ≥2, or ≥3 mm bone loss were 68%, 32%, and 10%, respectively. A multilevel growth curve model revealed that the bone loss model was not linear and demonstrated an increase in variance over time that was attributed to the heterogeneity of the subjects.

Interestingly, there was no correlation between the total area of the defect and sites regenerated with GBR techniques. Similarly, a systematic review by Salvi et al. [14] did not reveal any differences in the prevalence of peri-implantitis involving implants inserted in the native and regenerated bone.

One limitation of the study consists of the use of 2D intraoral radiographs: such radiographs exhibit some grade of geometric distortion, they do not give any information about the pattern of bone resorption on the bucco-lingual dimension, they do not allow the distinction between buccal and lingual plates [15], and, because of the superimposition of anatomical structures, may have masked the real maximum depth of the defect and the marginal bone levels. Although cone beam computed tomography (CBCT) is able to provide more information about the configuration and extension of the peri-implant defect in all three dimensions, intraoral radiographs exhibited higher accuracy compared to CBCT for the measurement of its mesiodistal width, as recently demonstrated by Steiger-Ronay et al. [16]. Actually, in their experimental setting, CBCT always led to an overestimation of the known defect width, especially when zirconium dioxide implants were considered [16].

In any case, it must be noted that the use of intraoral radiographs in the present study is linked to its retrospective design, since periapical radiographs are routinely used in daily practice to monitor the health of peri-implant hard tissues, while 3D imaging is used in more complex cases where information about the bucco-lingual dimension is needed.

Assessment of the bone level in implants with buccal defects remains problematic, and data from intraoral radiographs tend to overestimate the bone anchoring of these implants [17]. It has been observed that intraoral radiographs can show a resolution of 10–25 line pairs per mm, panoramic images show 3–5 line pairs, and CBCT only 1–2. In fact, the highest likelihood ratios were found for intraoral radiographs, indicating the best performance for intraoral radiographs in detecting peri-implant bone defects, while the lowest specificity was found with CT [18].

The amount of keratinized mucosa was not considered, which is crucial for its role as a coronal seal and in maintaining hygiene. Prosthetic misfit has not been considered in the present study. Although it can be hypothesized that prosthetic misfit may lead to a higher prevalence of peri-implantitis, a recent review by Katsoulis et al. [19] concluded that the association between prosthetic misfit and a higher rate of biological complications cannot be confirmed due to currently scarce information on this topic. Surprisingly, a recent analysis of 193 implants with peri-implant defects found that implant-abutment misfit was associated with slight peri-implant bone loss (2–3 mm). The authors explained these results, hypothesizing that the presence of prosthetic misfit could have actually been the peri-implant bone resorption, but once a certain distance has been established between the prosthetic gap and the marginal bone, the misfit itself stops contributing to further disease progression [20].

The primary limitation of this study is the small sample size. Future research with a larger sample size would be valuable to validate our findings in this study.

In conclusion, the results from this 2D radiographic study showed the prevalence of crateriform peri-implant defects, with a hygiene-correlated extension, perceptible on the mesial and distal aspects. Since 3D imaging of the peri-implant tissues is not part of the clinical routine and is not always available, our description does not take into consideration bone defects that are not appreciable on 2D examinations, such as dehiscence.

Further studies, possibly based on 3D imaging, are desirable to investigate any correlations that did not emerge in this analysis.

## Figures and Tables

**Table 1:** Radiographic measures of the peri-implant defects and results of the time from implant placement to peri-implantitis diagnosis.

Parameter	Mean ± standard deviation	95% CI
Mesial area (mm^2^)	7.8 ± 8.31	5.84–9.75
Distal area (mm^2^)	7.31 ± 8.53	5.31–9.31
Total area (mm^2^)	15.11 ± 15.94	11.36–18.85
Maximum radius	5.44 ± 1.71	5.04–5.84
Mesial depth	4 ± 2.28	3.46–4.43
Distal depth	3.92 ± 2.15	3.42–4.42
Mesial width	2.37 ± 1.55	1.99–2.74
Distal width	2.19 ± 1.63	1.8–2.58
Distance from the mesial surface	6.61 ± 3.17	5.73–7.49
Distance from the distal surface	5.97 ± 3.37	4.91–7.03
Residual mesial bone	2.72 ± 1.79	2.25–3.21
Residual distal bone	2.62 ± 1.69	2.11–3.14
Years from surgery to diagnosis of peri-implantitis	7.9 ± 3.13	7.19–8.64

**Table 2:** Demographic data, patient-related parameters, and implant-related parameters (patient-level and implant-level).

Parameter	Result (patient-level) (*n* = 27)	Result (implant-level) (*n* = 73)
Age at surgery (years)	—	56.5 ± 7.84 (24–69)
Sex	M: 13; F: 14	M: 30; F: 43
Smoking		
Smokers	12 (44.4%)	32 (43.8%)
Nonsmokers	15 (55.6%)	41 (56.2%)
Diabetes	1 (3.7%)	3 (4.1%)
History of periodontitis	17 (63.0%)	45 (61.6%)
Hygiene level		
Good	4 (14.9%)	14 (19.2%)
Fair	12 (44.4%)	22 (45.2%)
Poor	11 (40.7%)	26 (35.6%)
Recall frequency		
6 months	15 (55.6%)	43 (58.9%)
4 months	7 (25.9%)	13 (17.8%)
3 months	5 (18.5%)	15 (20.5%)
Implant brand		
NobelBiocare™	—	70 (95.9%) (Speedy Groovy™: 55 (75.3%); Replace™: 12 (16.4%); Branemark: 3 (4.1%))
3i	—	3 (4.1%)
Prosthetic rehabilitation		
AllOn4®	—	41 (56.2%)
Fixed partial prosthesis	—	23 (31.5%)
Single crown	—	9 (12.3%)
Implant length		
13 mm	—	25 (34.2%)
15 mm	—	21 (28.8%)
10 mm	—	14 (19.2%)
11.5 mm	—	12 (16.4%)
8.5 mm	—	1 (1.4%)
Implant diameter		
≤3.5 mm	—	8 (10.9%)
>3.5 and <5 mm	—	64 (87.7%)
≥5 mm	—	1 (1.4%)
Prosthetic retention		
Screw	—	61 (83.6%)
Cemented	—	11 (15.1%)
Regeneration	—	20 (27.4%)
Implant position		
Upper anterior	—	15 (20.6%)
Lower anterior	—	10 (13.7%)
Upper molar and premolar area	—	26 (35.6%)
Lower molar and premolar area	—	22 (30.1%)

Continuous variables are presented in terms of mean ± standard deviation (minimum—maximum). Discrete variables are presented in terms of absolute value (percentage).

## Data Availability

Data can be requested from the corresponding author.

## References

[1] Schwarz F., Herten M., Sager M., Bieling K., Sculean A., Becker J. (2007). Comparison of naturally occurring and ligature-induced peri-implantitis bone defects in humans and dogs. *Clinical Oral Implants Research*.

[2] Schwarz F., Sahm N., Schwarz K., Becker J. (2010). Impact of defect configuration on the clinical outcome following surgical regenerative therapy of peri-implantitis. *Journal of Clinical Periodontology*.

[3] Serino G., Turri A., Lang N. P. (2013). Probing at implants with peri-implantitis and its relation to clinical peri-implant bone loss. *Clinical Oral Implants Research*.

[4] García-García M., Mir-Mari J., Benic G. I., Figueiredo R., Valmaseda-Castellón E. (2016). Accuracy of periapical radiography in assessing bone level in implants affected by peri-implantitis: a cross-sectional study. *Journal of Clinical Periodontology*.

[5] Monje A., Insua A., Rakic M., Nart J., Moyano-Cuevas J. L., Wang H.-L. (2018). Estimation of the diagnostic accuracy of clinical parameters for monitoring peri-implantitis progression: an experimental canine study. *Journal of Periodontology*.

[6] Serino G., Turri A. (2011). Extent and location of bone loss at dental implants in patients with peri-implantitis. *Journal of Biomechanics*.

[7] Monje A., Pons R., Insua A., Nart J., Wang H.-L., Schwarz F. (2019). Morphology and severity of peri-implantitis bone defects. *Clinical Implant Dentistry and Related Research*.

[8] Berglundh T., Armitage G., Araujo M. G. (2018). Peri-implant diseases and conditions: consensus report of workgroup 4 of the 2017 world workshop on the classification of periodontal and peri-implant diseases and conditions. *Journal of Clinical Periodontology*.

[9] Karoussis I. K., Salvi G. E., Heitz-Mayfield L. J. A., Brägger U., Hämmerle C. H. F., Lang N. P. (2003). Long-term implant prognosis in patients with and without a history of chronic periodontitis: a 10-year prospective cohort study of the ITI® dental implant system. *Clinical Oral Implants Research*.

[10] Roos-Jansåker A.-M., Renvert H., Lindahl C., Renvert S. (2006). Nine-to fourteen-year follow-up of implant treatment. Part III: factors associated with peri-implant lesions. *Journal of Clinical Periodontology*.

[11] Koldsland O. C., Scheie A. A., Aass A. M. (2011). The association between selected risk indicators and severity of peri-implantitis using mixed model analyses. *Journal of Clinical Periodontology*.

[12] Roccuzzo M., Bonino F., Aglietta M., Dalmasso P. (2012). Ten-year results of a three arms prospective cohort study on implants in periodontally compromised patients. Part 2: clinical results. *Clinical Oral Implants Research*.

[13] Fransson C., Wennström J., Tomasi C., Berglundh T. (2009). Extent of peri-implantitis-associated bone loss. *Journal of Clinical Periodontology*.

[14] Salvi G. E., Cosgarea R., Sculean A. (2016). Prevalence and mechanisms of peri-implant diseases. *Journal of Dental Research*.

[15] Ritter L., Elger M. C., Rothamel D. (2014). Accuracy of peri-implant bone evaluation using cone beam CT, digital intra-oral radiographs and histology. *Dentomaxillofacial Radiology*.

[16] Steiger-Ronay V., Krcmaric Z., Schmidlin P. R., Sahrmann P., Wiedemeier D. B., Benic G. I. (2018). Assessment of peri-implant defects at titanium and zirconium dioxide implants by means of periapical radiographs and cone beam computed tomography: an in-vitro examination. *Clinical Oral Implants Research*.

[17] Schliephake H., Wichmann M., Donnerstag F., Vogt S. (2003). Imaging of periimplant bone levels of implants with buccal bone defects. *Clinical Oral Implants Research*.

[18] Kühl S., Zürcher S., Zitzmann N. U., Filippi A., Payer M., Dagassan-Berndt D. (2016). Detection of peri-implant bone defects with different radiographic techniques—a human cadaver study. *Clinical Oral Implants Research*.

[19] Katsoulis J., Takeichi T., Sol Gaviria A., Peter L., Katsoulis K. (2017). Misfit of implant prostheses and its impact on clinical outcomes. Definition, assessment and a systematic review of the literature. *European Journal of Oral Implantology*.

[20] Wehner C., Bertl K., Durstberger G., Arnhart C., Rausch-Fan X., Stavropoulos A. (2021). Characteristics and frequency distribution of bone defect configurations in peri-implantitis lesions—a series of 193 cases. *Clinical Implant Dentistry and Related Research*.

